# Pneumothorax, pneumomediastinum and subcutaneous emphysema following closed percutaneous pleural biopsy: a case report

**DOI:** 10.1186/1757-1626-1-274

**Published:** 2008-10-25

**Authors:** KB Sriram, HPA Jersmann

**Affiliations:** 1 Department of Thoracic Medicine, Royal Adelaide Hospital, Adelaide, South Australia 5000, Australia

## Abstract

Minimally invasive investigations, such as pleural fluid cytological assessment and closed percutaneous pleural biopsy, are often performed first in the investigation of suspected malignant pleural effusions. Malignant pleural effusions can be diagnosed with pleural fluid cytology alone in most cases; however, closed pleural biopsy is performed to increase the diagnostic yield when pleural fluid cytology is negative. This additional yield is at the expense of increased complication rates. We report a 64-year old man with a negative pleural fluid cytology but suspected malignant pleural effusion who underwent a closed pleural biopsy, which was complicated by pneumothorax, pneumomediastinum and severe subcutaneous emphysema. Pulmonary laceration by the pleural biopsy needle is the most likely aetiology of these complications. Our case report highlights an infrequent but significant complication of closed percutaneous pleural biopsy.

## Background

Malignant pleural effusions typically indicate disseminated disease, with an expected median survival of 3–12 months [1,2]. Prognosis is particularly poor when the pleural effusion is due to primary lung cancer [1,2]. There are several techniques available to investigate suspected malignant pleural effusions, ranging from simple procedures, such as pleural fluid aspiration and closed percutaneous pleural biopsy (CPB), to more complicated procedures, such as thoracoscopy. Guidelines for the diagnosis of unilateral pleural effusions typically recommend using minimally invasive investigations to obtain a diagnosis when malignancy is suspected [1]. We report a patient with an exudative unilateral pleural effusion who underwent a CPB and suffered a severe complication of this procedure.

## Case report

**Figure 1:**
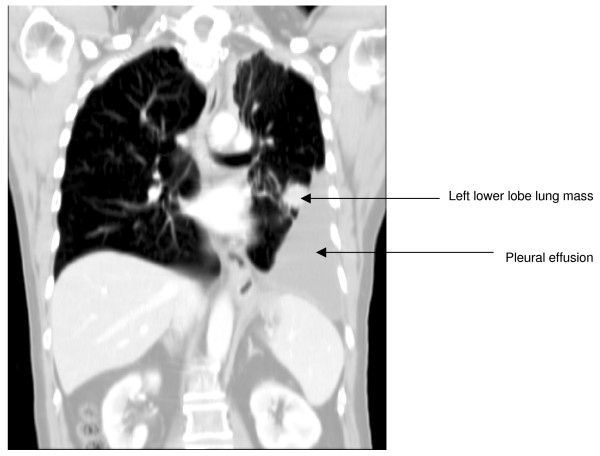
Coronal view of thoracic computed tomography scan showing large left pleural effusion and mass in the left lower lobe.

A 64-year-old-man was referred to our institution for investigation of a large left pleural effusion. Over the preceding two months, he had developed progressive dyspnea, left pleuritic chest pain and 6 kg weight loss. He was a reformed smoker with a 40-pack-year smoking history. He was admitted to the hospital, and a thoracic CT scan (Figure 1) was performed which showed a large left pleural effusion, left lung collapse and a left lower lobe mass. Pleural fluid cytology did not contain any malignant cells. Subsequently, CPB using Abrams needle was performed by a senior medical officer. Three out of four specimens contained normal pleural tissue without any malignant features.

**Figure 2:**
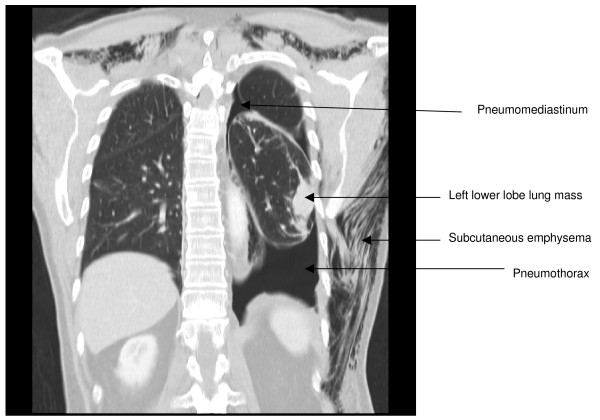
Coronal view of thoracic computed tomography scan showing pneumothorax, pneumomediastinum and subcutaneous emphysema which developed after iatrogenic pulmonary injury caused by Abrams pleural biopsy needle.

Twelve hours after the procedure, he developed a high pitched dysphonia, severe dyspnea, and pleuritic left chest pain. On examination, there was extensive subcutaneous emphysema along the chest wall extending to the neck. An emergency thoracic CT scan (Figure 2) was performed, which revealed subcutaneous emphysema, pneumothorax and pneumomediastinum. An intercostal chest tube was inserted, resulting in complete resolution of the pneumothorax. The subcutaneous emphysema, dysphonia and pneumomediastinum were resolved in seven days. The chest tube was successfully removed after seven days.

Subsequently, a CT guided FNA of the left lower lobe mass was obtained. This revealed non-small cell lung cancer, subtype adenocarcinoma. The patient is currently receiving palliative chemotherapy with symptomatic improvement and no recurrence of the pleural effusion.

## Discussion

Malignant pleural effusions can be diagnosed by pleural fluid cytology alone in 60% of cases, and CPB can increase the diagnostic yield by 7%–27% [1]. Pleural biopsies are performed using either an Abrams' or Cope needle and can be successfully performed with limited training [[1,3], and [4]]. Pneumothorax develops in 3–15% of patients undergoing CPB, and of these patients, 1% requires chest tube drainage [1,3,4]. Pneumomediastinum and subcutaneous emphysema after CPB has not been commonly reported.

Pneumothorax most commonly develops due to entry of air through the biopsy needle or laceration to the lung parenchyma. We hypothesize that in our patient, laceration to the lung parenchyma rather than air entry through biopsy needle is the most likely pathogenetic mechanism. The injury probably produced a bronchopleural fistula, allowing air to track along the continuum between the endothoracic fascia of outer chest wall, pleural space and mediastinum, resulting in subcutaneous emphysema, pneumothorax and pneumomediastinum [5]. We believe that air in the subcutaneous tissues of the neck caused the patient's dysphonia, because as the subcutaneous emphysema resolved so did the dysphonia.

Because of the limited yield and risk of complications, CPB has been superseded by thoracoscopy in some centers. Thoracoscopy is diagnostic in 95% of malignant pleural effusions [6,7]. The high yield is due to the direct visualisation of the pleura and the ability to obtain large biopsy specimens from abnormal pleura. Additionally, thoracoscopy can be used to perform talc pleurodesis, producing effective symptom palliation [6,7]. However, the procedure is expensive, requires highly trained operators and is not widely available.

Our report illustrates the complications of pneumothorax, pneumomediastinum and subcutaneous emphysema after a non-diagnostic CPB. We believe that by being aware of the limitations of CPB, clinicians can better choose the appropriate test in the investigation of exudative unilateral pleural effusions.

## Consent

Written informed consent was obtained from the patient for publication of this case report and accompanying images. A copy of the written consent is available for review by the Editor-in-Chief of this journal.

## Competing interests

The authors declare that they have no competing interests.

## Authors' contributions

KBS reviewed the case notes and prepared the manuscript. HPAJ read and approved the final manuscript.

## References

[B1] Maskell NA, Butland RJA (2003). BTS guidelines for the investigation of a unilateral pleural effusion in adults. Thorax.

[B2] Antunes G, Neville E, Duffy J, Ali N (2003). BTS guidelines for the management of malignant pleural effusions. Thorax.

[B3] Chakrabarti B, Ryland I, Sheard J, Warburton CJ, Earis JE (2006). The roles of Abrams percutaneous pleural biopsy in the investigation of exudative pleural effusions. Chest.

[B4] Baumann MH (2006). Closed Pleural Biopsy: Not Dead Yet!. Chest.

[B5] Murray, Nadel's (2005). Textbook of Respiratory Medicine.

[B6] Lee P, Colt HG (2005). Rigid and semirigid pleuroscopy: The future is bright. Respirology.

[B7] Rodriguez-Panadero F, Janssen JP, Astoul P (2006). Thoracoscopy: general overview and place in the diagnosis and management of pleural effusion. Eur Respir J.

